# Supplementary material for: Splice-Junction-Based Mapping of Alternative Isoforms in the Human Proteome
Source: Cell Rep. Author manuscript; Available in PMC 2020 Jan 15. (PMC6961840; doi:10.1016/j.celrep.2019.11.026)

A

sp|P27816|MAP4_HUMAN|ENSG00000047849|MXE2|2188|chr3|47917174|47918841|−2|r169|T1
AGQNDPLKDSYVPLELAK q value: 0.00033717 Tr_novel:TRUE RefSeq_Novel:FALSE
Search result spec prec mz: 979.5184 Actual spec prec mz: 979.51843
Fragments matched per AA: 2.78 Proportion of top 20 peaks matched: 0.4

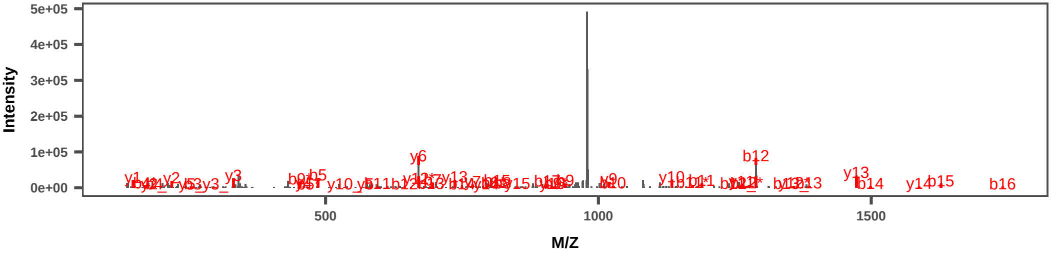

B

Scatterplot of predicted elution time
Fitting R2: 0.752
Novel peptide residual Z score: 0.132
Number of peptides: 3612

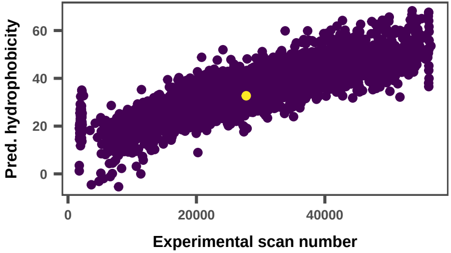

C

Distributions of residuals from best−fit line
of predicted RT vs Expt. scan number
Line: Z score of novel peptide
Z: 0.132

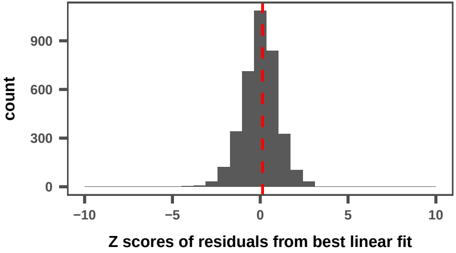

Supplement: 2 [file NIHMS1546469-supplement-2.zip › DF1/PXD010154/Testis/Testis_24_MAP4_AGQNDPLKDSYVPLELAK.pdf]
